# High-affinity CD16-polymorphism and Fc-engineered antibodies enable activity of CD16-chimeric antigen receptor-modified T cells for cancer therapy

**DOI:** 10.1038/s41416-018-0341-1

**Published:** 2018-11-15

**Authors:** Felicitas Rataj, Severin J. Jacobi, Stefan Stoiber, Florian Asang, Justyna Ogonek, Nicholas Tokarew, Bruno L. Cadilha, Erwin van Puijenbroek, Constanze Heise, Peter Duewell, Stefan Endres, Christian Klein, Sebastian Kobold

**Affiliations:** 1 Center of Integrated Protein Science Munich (CIPS-M) and Division of Clinical Pharmacology, Department of Medicine IV, Klinikum der Universität München, LMU Munich (Member of the German Center for Lung Research (DZL), LMU Munich, Germany; 2 Roche Innovation Center Zurich, Schlieren, Switzerland

**Keywords:** Immunotherapy, Tumour immunology

## Abstract

**BACKGROUND:**

CD16-chimeric antigen receptors (CAR) T cells recognise the Fc-portion of therapeutic antibodies, which can enable the selective targeting of different antigens. Limited evidence exists as to which CD16-CAR design and antibody partner might be most effective. We have hypothesised that the use of high-affinity CD16 variants, with increased Fc-terminus antibody affinity, combined with Fc-engineered antibodies, would provide superior CD16-CAR T cell efficacy.

**METHODS:**

CD16-CAR T (wild-type or variants) cells were co-cultured with Panc-1 pancreatic cancer, Raji lymphoma or A375 melanoma cells in the presence or absence of anti-CD20, anti-MCSP, wild-type or the glycoengineered antibody variants. The endpoints were proliferation, activation, and cytotoxicity in vitro.

**RESULTS:**

The CD16 158 V variant of CD16-CAR T cells showed increased cytotoxic activity against all the tested cancer cells in the presence of the wild-type antibody directed against MCSP or CD20. Glycoengineered antibodies enhanced CD16-CAR T cell activity irrespective of CD16 polymorphisms as compared with the wild-type antibody. The combination of the glycoengineered antibodies with the CD16-CAR 158 V variant synergised as seen by the increase in all endpoints.

**CONCLUSION:**

These results indicate that CD16-CAR with the high-affinity CD16 variant 158 V, combined with Fc-engineered antibodies, have high anti-tumour efficacy.

## Background

Adoptive T cell therapy (ACT) employs autologous T cells, which are expanded in vitro and therapeutically reinfuse into patients for the treatment of cancer. Clinically, three main avenues have been investigated: (1) tumour infiltrating lymphocytes (TIL) isolated from the patient´s tumour; (2) peripheral blood T cells transduced with T cell receptors (TCR) specific for cancer-associated antigens; and (3) peripheral blood T cells transduced with synthetic receptors, the so called chimeric antigen receptors (CARs).^1,2^ All three strategies have and are being investigated in clinical trials with varying success rates.^3^ The most advanced clinical strategies are CAR T cells specific for the B-cell differentiation antigen CD19. Various anti-CD19 CAR T cells have been evaluated in refractory B-cell malignancies, including acute lymphatic leukaemia (ALL) and diffuse large B-cell lymphoma (DLBCL). Based on previously unseen response rates, the prolongation of disease-free and overall survival, anti-CD19 CAR T cell therapies have been approved by the Food and Drug Administration (FDA) in 2017.^4,5^

In spite of these achievements, recent data also demonstrate that most patients are unlikely to benefit from these treatments in the long run and will relapse. Only a fraction of treated patients have a long-term benefit from CAR T cells or can be considered cured.^6^ Causes for primary or secondary resistance and treatment failure to CAR T cell therapy include: (1) loss or downregulation of the target antigen, resulting in insufficient cancer cell recognition;^7^ (2) insufficient access of the CAR T cells to the vicinity of the malignant cell—an aspect of particular relevance in the context of solid cancer treatment,^8^ and (3) local and systemic immune suppression, which impedes CAR T cells activity.^9^ These limitations will have to be overcome to broaden the applications of CAR T cell technology across several clinical indications. Access of T cells and circumvention of immune suppression will be of utmost importance when applying CAR-T cells to patients with solid cancers.

The first of these hurdles—loss, downregulation, or mutation of tumour-associated antigen^7,9,10^—could be addressed by the simultaneous or sequential targeting of different antigens. Given the manufacturing and regulatory burden to develop and produce a specific CAR T cell product, targeting different antigens with one CAR T cell product would bear considerable advantage.

A promising avenue is the use of CARs not based on a single-chain antibody variable fragment (for direct antigen recognition) but on the activating Fc-receptor CD16.^11^ This platform requires co-administration of monoclonal antibodies and CD16 CAR T cells for cancer recognition. This has two major advantages: it allows the use of already approved antibodies for tumour targeting, and it enables a target-switch (in the same patient, with the same T cell product) under therapeutic pressure.

Currently, CD16-CAR T cells are under pre-preclinical and clinical testing, in combination with monoclonal antibodies such as rituximab. First clinical trials have been initiated combining the CD20-targeting antibody rituximab with a CD16-CAR T cell product (ClinicalTrials.gov Identifier: NCT03189836).

CD16 has polymorphisms distributed within the normal population, which could affect the affinity for the Fc-part of antibodies. We hypothesised that altering these sequences in a CD16-CAR would also modulate CAR T cell activity. We also applied antibody engineering, to promote activity of CD16-CAR T cell by increasing Fc-binding affinity via CD16. We postulated that both strategies might enhance CD16-CAR T cell activity, and their combination might further benefit tumour cell recognition and lysis (Supplementary Figure 1A).

We have demonstrated that the high-affinity CD16 158 V variant strongly promotes CD16-CAR T cell activity in combination with both wild-type and Fc-engineered antibodies. Glycoengineered antibodies with enhanced FcγRIIIa affinity further increase tumour cell recognition and CD16-CAR T cell activation. Furthermore, we have demonstrated the value of the strategy by using the approved Fc-glycoengineered clinical-grade anti-CD20 antibody GA101 (obinutuzumab),^12^ the anti-MCSP antibody LC007 and their non-glycoengineered counterparts, in human B-cell lymphoma and melanoma cell lines, respectively.

## Materials and methods

### Cell lines

Raji, a human Burkitt lymphoma cell line, was purchased from the American Type Culture Collection (ATCC). Raji cells were cultured in RPMI (RPMI 1640; Lonza, Switzerland) supplemented with 10% foetal bovine serum (FBS, Life Technologies, USA), 100 U/ml penicillin and streptomycin (PS), and 2 mM L-glutamine, 1 mM sodium pyruvate (all from PAA, Germany), and 1 mM HEPES (Sigma-Aldrich, Germany). Panc-1, a human pancreatic cancer cell line, was purchased from the European collection of cell cultures (ECACC). A375, a human melanoma cell line, was previously described.^13^ Panc-1 and A375 cells were cultured in DMEM3 + (DMEM with 10% FBS, 100 U/ml PS, and 2 mM L-glutamine). The retroviral amphitropic packaging cell line Platinum A was purchased from Cell Biolabs (USA). DMEM medium for Platinum A cells additionally contained 10 µg/ml puromycin and 1 µg/ml blasticidin (both from Sigma-Aldrich, Germany). The cell lines used in experiments were regularly checked for mycoplasma with the MycoAlert^TM^ kit (Lonza, Basel, Switzerland). Primary human T cells were cultured in VLE-RPMI 1640 (Biochrom, Germany) supplemented with 2.5% human serum (Sigma-Aldrich, Germany), 100 U/ml PS and 2 mM L-glutamine, 1 mM sodium pyruvate, 10 µM NEAA (Sigma-Aldrich, Germany), and 50 µM β-mercaptoethanol (human TCM).

### Antibody generation

Antibodies cetuximab (Merck, Darmstadt, Germany), panitumumab (Amgen, Munich, Germany), and rituximab (Roche, Basel, Switzerland) were commercially purchased. Obinutuzumab (GA101) is a glycoengineered Type II anti-CD20 antibody that mediates enhanced cell death induction as compared with rituximab.^12^ Glycoengineered GA101 is produced in CHO cells co-expressing the glycosylating enzymes GnT3 and Man2.^14^ For comparative analysis, non-glycoengineered wild-type GA101 expressed in conventional CHO cells was used. As a negative control, we used a mutant version of GA101 which was produced by transient expression in HEK293 cells. The mutant version of GA101 contains P329G LALA mutations that abolished FcγR binding.^15^

For MCSP targeting, the humanised antibody LC007 M4-3 ML2 and the respective glycoengineered MCSP antibody LC007 M4-3 ML2-g2 were used. Both antibodies were produced by transient expression in HEK293 cells, either in the presence or absence of GnT3 and Man2 co-expression, respectively.^16^ All antibodies were purified via protein A chromatography and an ion exchange or size exclusion polishing steps. Purity and monomeric state were confirmed by CE-SDS and analytical size exclusion chromatography, respectively, and identity was confirmed by mass spectrometry.

### Generation of T cell activating CD16-CAR

The constructs were generated by overlap extension PCR and recombinant expression cloning into the retroviral pMP71 vector (kindly provided by C. Baum, Hannover). The CD16 fusion receptor constructs consist of the signal peptide (accession number NM_000569.6 aa 1–53) and extracellular domain of human CD16 (Uniprot Entry P 08637 aa 17–208), the transmembrane and intracellular domain of human CD28 (Uniprot Entry P10747 aa 153–220) and the intracellular domain of human CD3ζ (Uniprot Entry P20963 aa 52–164). The CD16 fusion receptor variants were generated by point mutation PCR in the CD16 extracellular domain as follows: aa 48 L → H and aa 158 F → V. The CD16del constructs are composed of the aforementioned signal peptide fused to CD16 molecule without the intracellular CD28 and CD3ζ signalling domain (Uniprot Entry P 08637 aa 17–229).

### Ethical approval and consent to participate

Use of PBMC samples from irreversibly anonymised healthy blood donors was approved by the local ethical committee.

### Human T cell transduction

Human T cell transduction was performed as previously described.^8,13^ In brief, the packaging cell line Platinum A (Cell Biolabs, USA) was seeded into six-well plates (Corning, Kaiserslautern, Germany) at varying cell numbers (0.8 –1.2 × 10^6^) and cultured overnight until they reached confluency of ~70–90%. Subsequently, Platinum A cells were transfected with 18 µg of DNA, using the calcium phosphate precipitation method. Cell culture medium was changed 6 h post transfection, to retain cell viability, and virus was harvested both 48 h and 72 h after transfection, respectively.

In parallel, human PBMC were isolated by density gradient separation (Biocoll Separating Solution, Biochrom, Germany), from heparinised whole blood of healthy donors. CD3^+^ cells were positively selected by MACS^®^ Technology (Miltenyi Biotec, Germany) and activated for 48 h on anti-CD3- and anti-CD28-coated beads (eBioScience, Frankfurt, Germany, clones HIT3a and CD28.2) in six-well plates, in human TCM supplemented with 200 U/ml IL-2 (Peprotech, Hamburg, Germany). For subsequent cultures, 5 ng/ml IL-15 (Peprotech, Hamburg, Germany) and Dynabeads^®^ Human T-Activator anti-CD3 anti-CD28 (Life Technologies, Darmstadt, Germany) were added. Twenty-four-well plates (Corning, Kaiserslautern, Germany) were coated for 24 h with 12.5 µg/ml of RetroNectin (TaKaRa Biotech, Japan) in PBS. Retrovirus from Platinum A cells was concentrated on the RetroNectin coated 24-well plates by centrifugation at 3000x g for 90 min at 32 °C, and activated T cells were added to virus-loaded plates at a concentration of 10^6^ T cells per ml human TCM per well. In total, two transduction hits were performed at 24 h intervals. T cells were then cultured in human TCM supplemented with IL-2, IL-15, and β-mercaptoethanol at a concentration of 10^6^ T cells/ml, with medium changes every 48 h. Transduction efficiency was assessed by staining for human CD16 (clone 3G8, BioLegend) (Supplementary Figure 2).

### Co-cultures of tumour cells and T cells

T cells were co-cultured with either Panc-1, Raji, or A375 tumour cells in a 10:1 effector to target cell ratio using 96-well flat bottom plates (Corning, Kaiserslautern, Germany). Medium was supplemented with different antibody concentrations, peripheral blood mononuclear cell (PBMCs) or polyclonal human immunoglobulins (IVIgs) (1 or 10 mg/ml, Privigen, CSL Behring, USA), as indicated in the respective figure. After 48 h of co-culture, supernatants were collected and release of human IFN-γ was assessed by ELISA (Becton Dickinson, Heidelberg, Germany).

To assess T cell proliferation, T cells were collected after 72 h of co-culture, an Fc-block with Human TruStain FcX™ (BioLegend) was applied and cells were stained with anti-human CD16, anti-human CD3 (clone HIT3a, BioLegend, USA), in addition to a live-dead stain (Zombie Aqua™ Fixable Viability Kit, BioLegend, USA). Equal amounts of Count Bright absolute counting beads (Life Technologies, USA) were added to each sample for absolute number quantification.

### Flow cytometry-based lysis assay

Raji cells were co-cultured for 24 h with T cells in a 96-well flat bottom plate (Corning, Kaiserslautern, Germany) in DMEM supplemented with 0.1 mM NEAA, 1 mM sodium pyruvate (Life Technologies, Darmstadt, Germany), and different antibodies (antibody concentration 1 µg/ml) in a ratio of 10:1. Prior to surface staining, Fc-block was performed using Human TruStain FcX™ (BioLegend, USA). Subsequently, cells were stained with anti-human CD19 (clone HIB19, BioLegend, USA), anti-human CD3 (clone HIT3a, BioLegend, USA), and anti-human CD16 (clone 3G8, BioLegend, USA) in PBS. A live-dead stain (Zombie Aqua™ Fixable Viability Kit, BioLegend, USA) was used to limit analysis to viable cells. Cell numbers were determined by Count Bright absolute counting beads (Life Technologies, USA).

### Real-time cytotoxicity assays

In total, 10.000 A375 cells were plated in 150 µl DMEM (Lonza, Basel, Switzerland) supplemented with 0.1 mM NEAA, 10% FBS, 100 μg/ml streptomycin, 1 IU/ml penicillin, 2 mM L-glutamine, and 1 mM sodium pyruvate (Life Technologies, Darmstadt, Germany) in an xCELLigence 96-well flat bottom plates (ACEA Biosciences, San Diego, USA). After overnight incubation T cells, in a 10:1 effector to target ratio, PBMCs in a 10:1 PBMC to T cell ratio and corresponding antibodies (10 µg/ml) as indicated in the figures, were added. T cell killing was determined as change in impedance every 6 min for 20 h and afterwards for 45 h every 15 min the followed by an additional 45 h every 15 min, by using the xCELLigence® RTCA SP device.

### Statistics

The FACS data were analysed with FlowJo V9.2 or V10.3 software. Statistical analysis was performed by using GraphPad Prism software 5.0 or 7.0. Differences between experimental conditions were analysed using the unpaired two-tailed Student’s *t* test, with *p*-values < 0.05 were considered significant. In the case of repeated testing of the same samples (longitudinal analysis over time), two-way ANOVA with Bonferroni-correction was used. Data are shown as mean values ± SEM of a minimum of three biological replicates or independent experiments, as indicated. Supplementary Table 1 contains all *p*-values associated with the figures.

## Results

### CD16 158 V/F, but not 48 H/L variants, enhance activity of CD16-CAR T cells

**Fig. 1:**
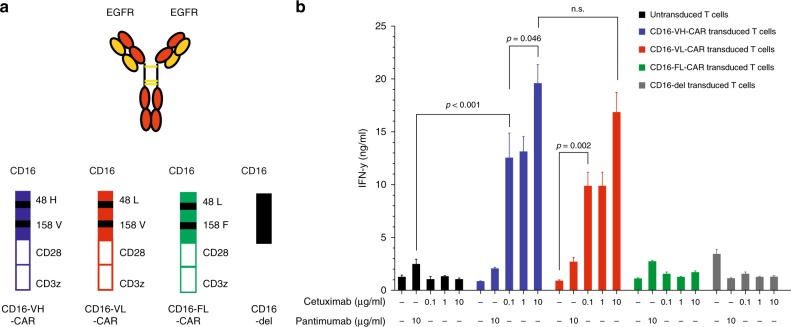
CD16 158 V but not 158 F or 48 H/L polymorphisms enable activity of CD16-CAR transduced T cells. **a** Schematic overview of used constructs; anti-EGFR monoclonal antibodies cetuximab (IgG1) and panitumumab (IgG2), CD16-CAR with 48 H and 158 V (referred to as VH), 48 L and 158 F (referred to as FL), 48 L and 158 V (referred to as VL) variations and CD16-del, which is devoid of the intracellular moieties. **b** In total, 300.000 CD16 VH-CAR-, CD16 VL-CAR-, CD16 FL-CAR-, CD16-del-transduced or untransduced T cells were co-cultured for 48 h with 30.000 EGFR^+^ Panc-1 pancreatic cancer cells in the presence or absence of 10 µg/ml of the anti-EGFR antibody panitumumab (IgG2) or increasing doses of the anti-EGFR antibody cetuximab (0.1, 1, and 10 µg/ml; IgG1), as indicated in the figure. IFN-γ production was measured by ELISA. All graphs show mean values of at least three technical replicate and each experiment shown is a representative figure of at least three independent experiments. A two-sided unpaired Student´s *t* test was used to determine the p-values and a p-value < 0.05 was considered statistically significant

To test the influence of CD16 polymorphisms on the activity of CD16-CAR T cells, we started by generating variants bearing sequential modifications that have been reported to enhance affinity of CD16 to the Fc-terminus of antibodies. We cloned and transduced the CD16-CARs 158 V 48 H, 158 V 48 L, and 158 F 48 L, as well as one construct without the signalling moieties (CD16-del, Fig. 1a). To test the impact of these receptors on T cell function, we took advantage of two approved anti-EGFR antibodies; the IgG1-based antibody cetuximab and the IgG2-based antibody panitumumab. The first would be expected to bind to CD16 and the latter not.

Primary human T cells bearing CD16-CAR, with the aforementioned structural changes were co-cultured with EGFR + Panc1 pancreatic cancer cells in the presence of either panitumumab or increasing concentrations of cetuximab. Cetuximab dose-dependently increased the activity of both the CD16 158 V 48H- and CD16 158 V 48L-CAR, and modestly CD16 158 F 48L-CAR (Fig. 1b). The CD16 48 H or 48 L variants had no influence on CD16-CAR function (Fig. 1b). As expected, co-culture in the presence of panitumumab did not induce IFN-y. These results demonstrate that the CD16 158 V construct enhances CD16-CAR T cell activity.

### Glycoengineered anti-CD20 antibodies enhance the activity of CD16-CAR T cells against CD20 + lymphoma cells, irrespective of CD16 variants

**Fig. 2:**
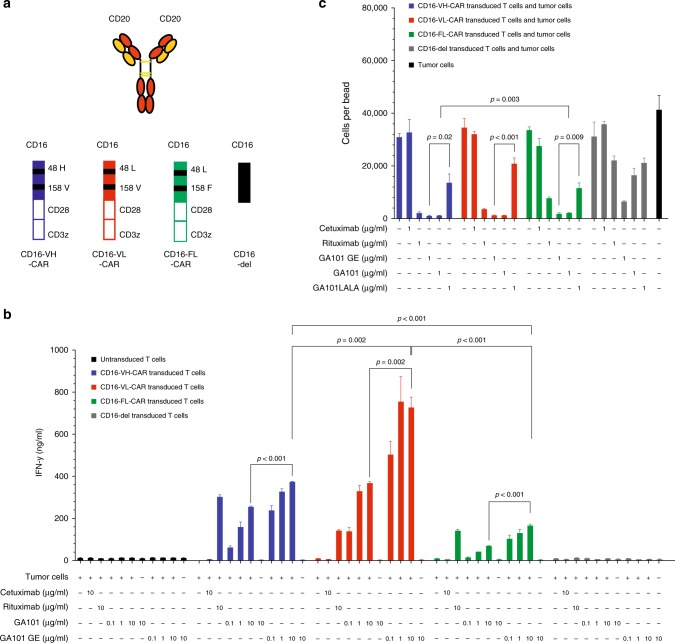
Glycoengineered anti-CD20 antibody GA101 enhances the activity of CD16-CARs against B-cell lymphoma cells. **a** Schematic overview of investigated constructs; anti-CD20 monoclonal antibodies (rituximab, GA101 or glycoengineered (GE) GA101), CD16-CAR with VH, FL, VL variants and Vdel. **b** 300.000 CD16 VH-CAR-, CD16 VL-CAR-, CD16 FL-CAR-, CD16-del-transduced or untransduced T cells were co-cultured for 48 h with 30.000 CD20^+^ Raji B-cell lymphoma cells in the presence or absence of either 10 µg/ml of the anti-EGFR antibody cetuximab (negative control), 10 µg/ml of the anti-CD20 antibody rituximab (positive control), increasing doses of the anti-CD20 antibodies GA101 or glycoengineered GA101GE (0.1, 1, and 10 µg/ml), as indicated in the figure. IFN-γ production was measured by ELISA. **c** CD16 VH-CAR-, CD16 VL-CAR-, CD16 FL-CAR-, CD16-del-transduced, or untransduced T cells were co-cultured for 24 h with 50.000 CD20 + Raji B-cell lymphoma cells in the presence or absence of either 1 µg/ml of the anti-EGFR antibody cetuximab, 1 µg/ml of the Fc-mutated anti-CD20 antibody GA101LALA (negative control), 1 µg/ml of the anti-CD20 antibody rituximab (positive control), 1 µg/ml of the anti-CD20 antibody GA101 or glycoengineered GA101 GE, as indicated in the figure. Cytotoxicity was assessed by flow cytometry by quantifying the absolute number of live Raji cells at the end of the assay. All graphs show mean values of at least three technical replicate and each experiment shown is a representative figure of at least three independent experiments. A two-sided unpaired Student´s *t* test was used to determine the *p*-values and a *p*-value < 0.05 was considered statistically significant

Next, we investigated the influence of the Fc-glycoengineered antibodies on the activity of CD16-CAR and its dependences on the CD16-CAR variants described above. We took advantage of the clinically approved anti-CD20 antibody GA101 (obinutuzumab), which is glycoengineered for about 10-fold enhanced CD16-binding (referred to as GE^12^). We generated a non-glycoengineered wild-type counterpart, which binds to the same epitope. We used another approved anti-CD20 antibody (rituximab) as a positive control as well as cetuximab as a negative control (Fig. 2a).

In T cell co-cultures with CD20^+^ Raji lymphoma cells, GA101GE enhanced T cell activation (as seen by the increase in IFN-γ release) via CD16-CAR more efficiently than the identical concentrations of wild-type GA101 or rituximab, regardless of the CD16-CAR variant (Fig. 2b). For both GA101 and GA101GE, activation of CD16-CAR was correlated positively with the dose of antibody used (Fig. 2b). Activation of the high-affinity CD16 158V-CAR T cells was, however, higher in the presence of all anti-CD20 antibodies, as compared with the low-affinity CD16 158F-CAR T cells (Fig. 2b), with the same antibody concentration. Furthermore, the combination of GA101GE with the CD16 48 L variant enhanced CD16-CAR T cell activation more efficiently than the CD16 48 H variant (Fig. 2b). In all cases, activation correlated with the dose of antibody used, and to a lesser extent depending on whether the CD16 158 V or CD16 158 F variant of the CAR was used.

All anti-CD20 antibodies induced lysis of lymphoma cells in a CD16-CAR T cell-dependent manner. CD16 158V-CAR-T cells were more effective at lysing CD20 + cells than CD16 158F-CAR-T cells in combination with rituximab (Fig. 2c), congruent to the findings for T cell activation in Fig. 2b. Vice versa, the use of an Fc-incompetent GA101 (GA101 LALA) prevented CD16-CAR-mediated lysis confirming that CD16 engagement is the mode of action of CD16-CAR T cells. Importantly the known non-Fc-mediated activity of GA101 on B-cell lymphoma viability was conserved in GA101 LALA conditions.^17^ For a given polymorphism, the activity of GA101 was higher than that seen with rituximab but was not further enhanced by glycoengineering, presumably because cell lysis was already maximal (Fig. 2c). We hereby provide evidence that co-administration of Fc-engineered antibodies may enhance the activity of CD16-CAR to target T cells against lymphoma cells.

### Glycoengineered anti-MCSP antibody synergises with the CD16 158 V variant for increased activation of CD16-CAR T cells and enhanced melanoma cell lysis

**Fig. 3:**
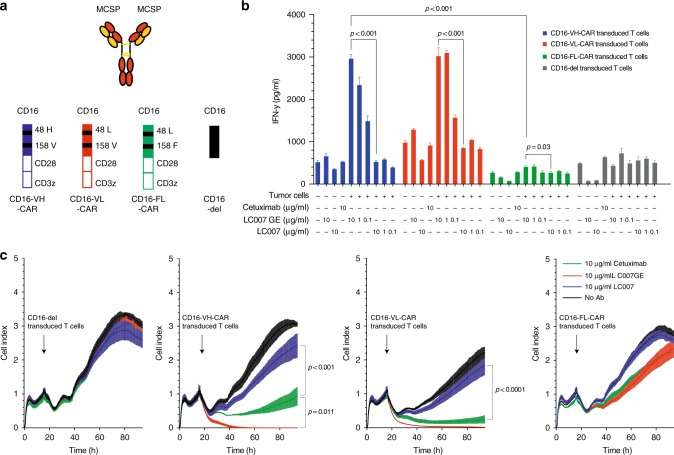
Glycoengineered anti-MCSP antibody LC007 synergises with CD16-158V-CAR for effective melanoma cell recognition and lysis. **a** Schematic overview of constructs used in this figure; the anti-MCSP antibody (LC007 or glycoengineered LC007GE), CD16-CAR with VH, FL, VL variants and Vdel. **b** CD16 VH-CAR-, CD16 VL-CAR-, CD16 FL-CAR-, CD16-del-transduced T cells were co-cultured for 48 h with 20.000 MCSP^+^ A375 melanoma cells in the presence or absence of either 10 µg/ml of the anti-EGFR antibody cetuximab (positive control), increasing doses of the anti-MCSP antibodies LC007 or increasing doses of the glycoengineered LC007GE (10, 1, and 0.1 µg/ml) as indicated in the figure. IFN-γ production was measured by ELISA. **c** 10^5^ CD16 VH-CAR-, CD16 VL-CAR-, CD16 FL-CAR- or CD16-del-transduced T cells were added after overnight incubation to 10^4^ MCSP^+^ A375 melanoma cells, in the presence or absence of either 10 µg/ml of the anti-EGFR antibody cetuximab, 10 µg/ml of the anti-MCSP antibody LC007 or glycoengineered LC007GE, as indicated in the figure. The time point when T cells and antibodies were added to the system are indicated by the black arrows. Cytotoxicity was measured in real-time, as a mean of cell viability by xCELLigence technology. All graphs show mean values of at least three technical replicate and each experiment shown is a representative figure of at least three independent experiments. For figure B a two-sided unpaired Student´s *t* test was used and for C a two-way ANOVA with Bonferroni-correction was used to determine the *p*-values. A *p*-value < 0.05 was considered statistically significant

To extend our findings on the relationship of CD16-CAR activity and affinity-driven binding of the Fc-portion of therapeutic antibodies, we next used the glycoengineered anti-MCSP antibody LC007 (LC007GE) and its non-engineered counterpart (LC007) (Fig. 3a). The aforementioned antibodies were tested in combination with the different CD16-CAR variants. Cetuximab was used as a positive control, on MCSP^+^EGFR^+^CD20^-^ A375 melanoma cells. Co-culturing CD16-CAR T cells with A375 cells in the presence of antibodies, resulted in a dose-dependent increase in activation for both anti-MCSP antibodies (Fig. 3b). Again, activation was highest for T cells transduced with CD16 158V-CAR variants, as compared with CD16 158F-CAR-T cells. For the anti-MCSP antibodies, we did not detect major differences in the activation capacity between co-incubated CD16 48H- and CD16 48L-CAR-transduced T cells (Fig. 3b). Activation was paralleled by induction of T cell proliferation by the LC007 GE antibody but not the wild-type LC007 antibody (Supplementary Figure 1B).

Quantifying the cytotoxic potential of CD16-CAR T cells in combination with the different antibodies, we found that all variants of CD16-CAR T cells induced cell lysis to a certain degree. However, only the CD16 158V-CAR-T cells (CD16 VH or CD16 VL) together with the glycoengineered LC007 mediated complete lysis of the melanoma cells, while the wild-type LC007 only led to transient growth control of tumour cells (Fig. 3c). These results indicate that glycoengineered antibodies with enhanced CD16 affinity and high-affinity CD16-CAR T cells synergise for maximal T cell efficacy against A375 melanoma cells.

### Presence of an excess of polyclonal human immunoglobulins and PBMCs modulates CD16-CAR T cell activation and tumour cell lysis

**Fig. 4:**
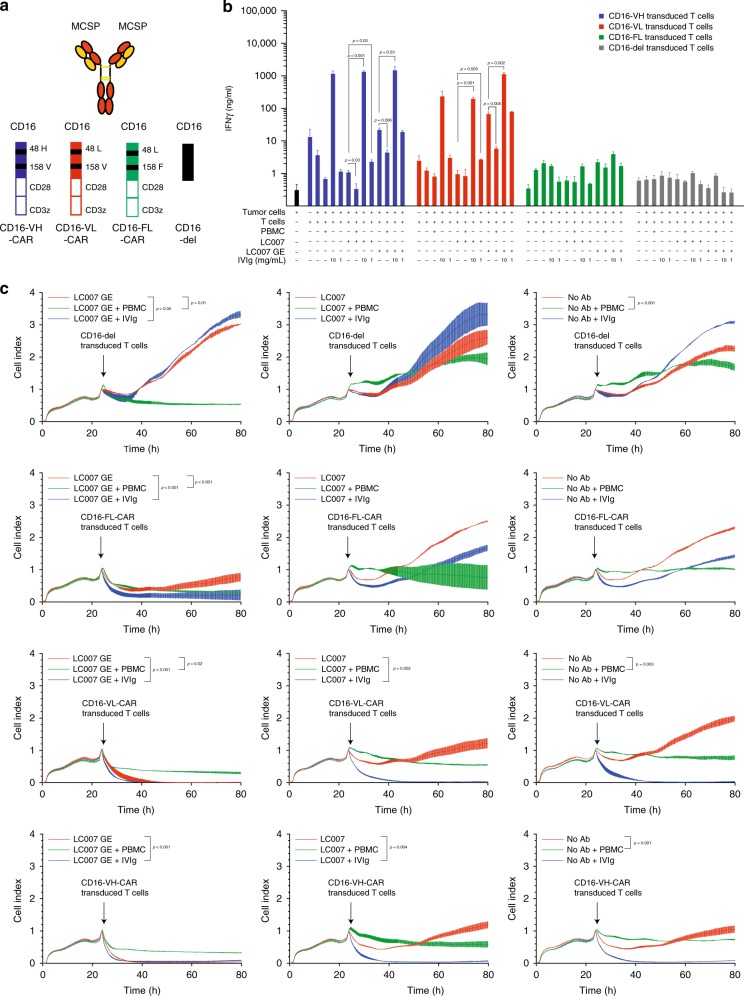
CD16-CAR T cell activation and cytoxicity in complex co-culture systems. **a** Schematic overview of constructs used in this figure; the anti-MCSP antibody (LC007 or glycoengineered LC007GE), CD16-CAR with VH, FL, VL variants and Vdel. **b** In total, 20 × 10^4^ CD16 VH-CAR-, CD16 VL-CAR-, CD16 FL-CAR-, CD16-del-transduced T cells were co-cultured for 48 h with 2 × 10^4^ MCSP^+^ A375 melanoma cells in presence of either 10 µg/ml of the anti-MCSP antibody LC007, 10 µg/ml of the anti-MCSP glycoengineered antibody LC007GE or without antibody. Each condition was additionally tested in the presence or absence of 10 mg/ml or 1 mg/ml polyclonal immunoglobulin or 2 × 10^6^ PBMC. IFN-γ production was measured by ELISA. **c** IN total, 10^5^ CD16 VH-CAR-, CD16 VL-CAR-, CD16 FL-CAR-, CD16-del-transduced T cells were added after overnight incubation to 10^4^ MCSP^+^ A375 melanoma cells, in presence of either 10 µg/ml of the anti-MCSP antibody LC007, 10 µg/ml anti-MCSP glycoengineered antibody LC007GE or without antibody. Each condition was additionally tested in the presence or absence of 10 mg/ml or 1 mg/ml polyclonal human immunoglobulins (IVIgs) or 10^6^ PBMC. The time point when T cells and antibodies were added to the system are indicated by arrows. Cytotoxicity was measured in real-time, as a mean of cell viability by xCELLigence technology. All graphs show mean values of at least three technical replicate and each experiment shown is a representative figure of at least three independent experiments. For figure B a two-sided unpaired Student's *t* test was used and for C a two-way ANOVA with Bonferroni-correction was used to determine the *p*-values. A *p*-value < 0.05 was considered statistically significant

As a patient is a complex organism as compared with an in vitro culture system composed of two or three components, several factors can affect CD16 CAR T cell activity, targeting, and safety. The components that are most likely to affect the efficacy and safety are immunoglobulins and other immune cells, which are able to engage the CD16 CAR T cells. To address these potential issues, we probed the impact of an excess of immunoglobulin or PBMCs on the activity of CAR T cells in the presence or absence of anti-MSCP antibody (LC007 or LC007GE, Fig. 4a) against A375 melanoma cells. In the context of wild-type LC007, the addition of PBMCs to the culture system has a modulating effect on T cell activation, as seen by interferon-γ release (Fig. 4b), and limits the cytotoxic activity of VL, FL, and VH CD16-CAR T cells (Fig. 4c). However, in the context of LC007GE, the addition of whole blood PBMCs to the culture system reduces target tumour cell lysis of VL, FL, and VH CD16-CAR T cells (Fig. 4c). This reduction in tumour cell killing in the presence of PBMCs is attributed to a reduction in T cell activation (Fig. 4b).

We further extended our exploration by adding polyclonal human antibodies to the aforementioned culture system. The addition of polyclonal human antibodies to the culture system dose-dependently enhanced T cell activation as compared with antibody alone, (Fig. 4b) and mediated a complete lysis of melanoma cells in the context of the high-affinity receptors CD16 VH- and CD16 VL-CAR-T cells (Fig. 4c). Concentrations of polyclonal human antibodies comparable with physiological serum concentrations (10 mg/ml) but not sub-physiological concentrations (1 mg/ml) induced on-tumour activation (Fig. 4b). Activation was paralleled by on tumour cell lysis by the CD16- CAR T cells (Fig. 4c). Addition of polyclonal human immunoglobulins did, however, not impede the efficacy of the different CD16-CAR variants (Fig. 4b, c).

## Discussion

We demonstrate that affinity modulating CD16 sequence variants play a major role in binding of antibodies by CD16-CAR T cells and can enhance their activity. The high-affinity 158 V variant renders CD16-CAR T cells superior to the more prevalent 158 F low-affinity variant of CD16-CAR T cells. Glycoengineering of the Fc-portion of antibodies for enhanced CD16 binding promotes both the activation and lytic activity of CD16-CAR T cells.

Our results provide evidence that both strategies enhance CD16 function, namely CD16s' own affinity for the Fc portion and engineering the Fc portion itself can be employed to promote T cell activity against cancer cells.

Several groups have previously reported the use of CD16-CAR T cells in preclinical models, including B- and T cell non-Hodgkin lymphoma, neuroblastoma, and breast cancer.^11^ Different designs have been used to trigger antibody-redirected cytotoxicity: in analogy to CAR, first (CD3ζ only) and second-generation (CD28 or CD137 and CD3ζ) designs have been utilised for the CD16-CAR structure.^18–20^ These CD16-CAR T cells represent a further development in respect to previously used CD16-transduced T cells.^21^ Importantly, no study has yet compared these designs with one another, and it is unclear if the chosen architectures are suited for optimal CD16-CAR T cell activity. The CD16-CAR reported in the present study represent, according to the above-referenced nomenclature, a second-generation CD16-CAR (i.e., containing domains CD28 and CD3ζ). Unlike previously published receptors, however, our transmembrane domain is a sequence of CD28 rather than that of CD3ζ or CD8.

By default, previous studies have employed the CD16 extracellular domain containing a 158 V mutation, yet, all authors postulate that any advantage seen with natural CD16 would also be transferrable to CD16-CAR. We now demonstrate that the 158 V variant indeed improves CD16-CAR T cell activity, as compared with the 158 F variant. Owing to the controversial relevance of CD16 variants for the activity of therapeutically applied antibodies, we argue that this comparison is of particular importance.^22^ The 48 H variant, although reported to enhance Fc affinity of CD16,^23^ did not influence CD16-CAR activity in our study.

Glycoengineering or affinity enhancing mutations of the Fc-portion have been applied to enhance binding of therapeutic antibodies to Fc-receptors and thereby to confer enhanced anticancer activity.^24,25^ Based on superior efficacy in comparison with rituximab, one such antibody—obinutuzumab—has been approved for the treatment of follicular lymphoma and chronic lymphatic leukaemia,^26^ indicating that CD20-targeting can be improved through Fc-engineering. As the activity of CD16 is subjected to Fc-binding and affinity, enhancement of CD16-CAR activity by Fc-glycoengineering of co-administered antibodies appeared probable. We demonstrate that antibodies against MCSP and CD20, when glycoengineered, conferred higher CD16-CAR T cell activity regardless of CD16 variants, this increase was more pronounced in the CD16 158 V variant.

Our data indicate that Fc-engineered antibodies can enhance CD16-CAR T cell activity. Approved or advanced clinical phase glycoengineered antibodies would be the first choice when designing clinical trials with CD16-CAR T cells in combination with antitumour antibodies. As exemplified in the present study, the glycoengineered anti-CD20 antibody obinutuzumab, enhances CD16-CAR T cell activity. Other candidates include the glycoengineered anti-CD19 antibody MOR208, which is under investigation in refractory B-cell non-Hodgkin lymphoma, and the glycoengineered anti-CCR4 antibody KW-0761, mogamulizumab, tested for T cell lymphoma.^27,28^ It is however important to bear in mind that enhanced activity, especially in the context of CAR T cells which can come with a severe side effect profile,^4^ carries the risk of more dismal side effects too. However, the clear advantage of CD16-CAR might come in handy here, as any CD16-CAR activity will vanish with antibody half-life and decay, this providing an inherent safety switch to this strategy.

In patients, endogenous polyclonal IgG molecules will compete with co-administered therapeutic antibodies for Fc-binding by CD16. Here, glycoengineered antibodies come with a clear advantage: They will be preferentially bound to CD16 compared with non-engineered antibodies. This has been shown for example, for rituximab versus obinutuzumab.^12^ In autoimmune diseases, where autoantibody titres occur, such discriminatory capacity can be of the particular importance:^29^ On the other hand, higher affinity autoantibodies might be able to trigger CD16-CAR T cells on their own right and this consideration might call for autoimmunity as an exclusion criteria for the use of CD16-CAR T cells. As a matter of fact, current studies with CD16-CAR specifically exclude these patients from enrolment. Glycoengineered antibodies can help CD16-CAR T cells to better discriminate between endogenous and therapeutically applied antibodies for enhanced efficacy and safety.

In order to further investigate safety of the proposed therapy, we probed the impact of high-dose polyclonal immunoglobulins and PBMCs. In this system when we cultured CD16 CAR T cells with 10 mg/ml of polyclonal human immunoglobulins, a concentrations found in the serum of healthy donors,^30^ we observed an increase in cytotoxicity and activation of enhanced affinity CD16 CAR T cells (VH and VL). Enhanced activity mediated by CD16-CAR in the presence of high doses of polyclonal immunoglobulins was due to the unspecific deposition of IVIgs on tumour cells, which subsequent activated the CAR T cells (data not shown). However, it is important to note that doses required for polyclonal triggering of CD16-CAR T cells were several log-concentrations higher than for monoclonal antibodies (microgram vs. milligram range). This question will be elucidated in ongoing clinical trials. In an in vivo context, this level of T cell activation could lead to autoimmunity or a cytokine storm,^31^ an event which may be enhanced in a “leaky” tumours, where high molecular weight serum components can extravasate and deposit in the tumours milieu.^32^ While in healthy tissues, the amount of deposited antibodies and higher molecular weight serum components should be negligible.^33^ These data would argue for the exclusion of patients with IgG-associated pathologies where antibodies can be deposited in tissues.^34,35^ Furthermore, our data would also argue for cautious T cell updosing to allow proper safety assessment in patients. In contrast, we observed that the addition of PBMC to our co-culture system inhibits cytotoxicity and activation of CD16 CAR T cells, what may counteract effect of polyclonal immunoglobulins in the peripheral blood and even prevent aforementioned pathologies. The observed dampening effect of PBMCs on the T cell response might have been caused by different subsets of PBMCs (e.g., regulatory T cells, myeloid derived suppressor cells) or alternatively, through competition over trophic factors. Moreover, aggregation of a high number of PBMC on the surface of cancer cells might have prevented the direct contact of effector T cells resulting in a decrease in the overall T cell activation level.

Different modular strategies have been employed to conditionally control T cell activity. For example, the “Uni-CAR”-platform employs a CAR recognising nanobody.^36^ Such approaches come with the advantage of being modular and bearing an inherent safety switch based on the antibodies' half-life. A similar concept has been utilised when monoclonal antibodies were tagged with FITC and targeted by an anti-FITC CAR T cell.^37^ However, all these strategies require both further antibody engineering of otherwise approved antibodies or modification and the development of cellular products. The major advantage of CD16-CAR T cells is the use of a single cellular product, irrespective of tumour-associated antigen to be targeted in combination with an approved monoclonal antibody. This combination has major regulatory advantages for development. The findings of this study may help to prioritise CD16-CAR design. It provides the rationale for the use of glycoengineered antibodies for optimal targeting of different types of cancer cells by CD16-CAR T cells.

## Electronic supplementary material

Supplementary Figure 1

Supplementary Figure 2

Supplementary Figure

## Data Availability

Materials and data available upon reasonable request.
