# Supplementary figures and images for: High-affinity CD16-polymorphism and Fc-engineered antibodies enable activity of CD16-chimeric antigen receptor-modified T cells for cancer therapy
Source: Br J Cancer. 2018 Nov 15;120(1):79–87. doi: 10.1038/s41416-018-0341-1 (PMC6325122; doi:10.1038/s41416-018-0341-1)

SUPPLEMENTARY FIGURE 1

A

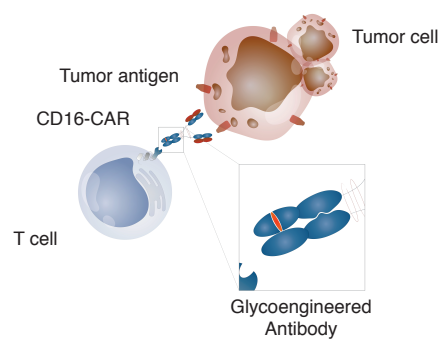

B

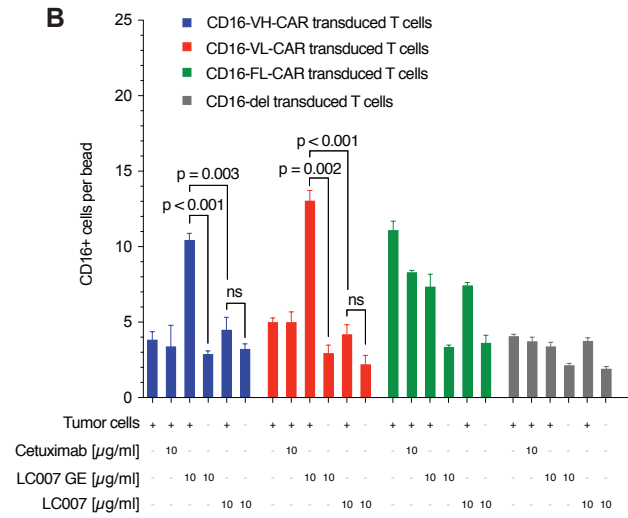

Supplement: Supplementary file 1 — Supplementary Figure 1 [file 41416_2018_341_MOESM1_ESM.pdf]

**SUPPLEMENTARY FIGURE 2**

**A**

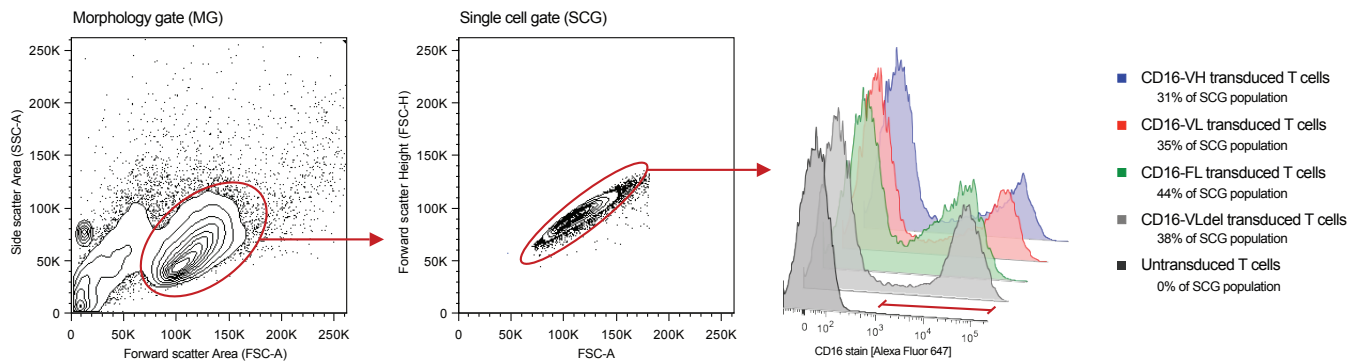

Supplement: Supplementary file 2 — Supplementary Figure 2 [file 41416_2018_341_MOESM2_ESM.pdf]
