# Supplementary material for: High-affinity CD16-polymorphism and Fc-engineered antibodies enable activity of CD16-chimeric antigen receptor-modified T cells for cancer therapy
Source: Br J Cancer. 2018 Nov 15;120(1):79–87. doi: 10.1038/s41416-018-0341-1 (PMC6325122; doi:10.1038/s41416-018-0341-1)
Supplement: Supplementary file 3 — Supplementary Figure [file 41416_2018_341_MOESM3_ESM.pdf]

Figure 1 B

	CD16-VH-CAR		CD16-VL-CAR		CD16-FL-CAR	
	p-value	fold diff.	p-value	fold diff.	p-value	fold diff.
no Ab vs [0.1] Cetuximab	0,01	14,11	0,003	8,37	>0,05	1,30
no Ab vs [1] Cetuximab	0,001	14,77	0,001	9,57	>0,05	1,05
no Ab vs [10] Cetuximab	<0,001	22,04	0,001	14,26	0,02	1,43
[0.1] Cetuximab vs [1] Cetuximab	>0,05	1,05	>0,05	1,14	>0,05	1,24
[0.1] Cetuximab vs [10] Cetuximab	>0,05	1,56	0,04	1,70	>0,05	1,09
[1] Cetuximab vs [10] Cetuximab	0,05	1,49	>0,05	1,49	0,03	1,36

	Panitumumab		Cetuximab w/o tumor cells		[0.1] Cetuximab		[1] Cetuximab		[10] Cetuximab	
	p-value	fold diff.	p-value	fold diff.	p-value	fold diff.	p-value	fold diff.	p-value	fold diff.
VL vs VH	>0,05	1,05	>0,05	1,30	>0,05	0,79	>0,05	0,86	>0,05	0,86

Figure 2 B

	CD16-VH-CAR		CD16-VL-CAR		CD16-FL-CAR	
	p-value	fold diff.	p-value	fold diff.	p-value	fold diff.
GA101 [10] vs Rituximab	0,002	1,24	<0,001	5,10	0,05	1,17
GA101 [0.1] vs GA101 GE [0.1]	0,002	3,8	0,005	3,61	0,007	6,91
GA101 [1] vs GA101 GE [1]	0,003	2,05	0,03	2,29	0,008	3,07
GA101 [10] vs GA101 GE [10]	<0,001	1,46	0,002	1,98	<0,001	2,38

	CD16-VH-CAR with Rituximab		CD16-VL-CAR with Rituximab	
	p-value	fold diff.	p-value	fold diff.
CD16-FL-CAR with Rituximab	<0,001	2,13	>0,05	1,00

	CD16-VL-CAR	
CD16-VH-CAR	p-value	fold diff.
GA101GE [0.1] vs GA101 GE [0.1]	0,02	2,12
GA101GE [1] vs GA101 GE [1]	0,02	2,30
GA101GE [10] vs GA101 GE [10]	0,002	1,94

Figure 2 C

	CD16-VH-CAR with GA101GE [1]		CD16-VL-CAR with GA101GE [1]		CD16-FL-CAR with GA101GE [1]	
	p-value	fold diff.	p-value	fold diff.	p-value	fold diff.
Rituximab	>0,05	2,09	<0,001	2,90	0,001	4,22
GA101 [1]	>0,05	1,11	>0,05	1,02	>0,05	1,20
GA101 LALA[1]	0,02	13,17	<0,001	16,60	0,009	6,33

	CD16-VH-CAR with GA101		CD16-VL-CAR with GA101		CD16-FL-CAR with GA101	
	p-value	fold diff.	p-value	fold diff.	p-value	fold diff.
Rituximab	>0,05	1,90	<0,001	2,90	>0,05	1,34

Figure 3B

	CD16-VH-CAR		CD16-VL-CAR		CD16-FL-CAR	
	p-value	fold diff.	p-value	fold diff.	p-value	fold diff.
no Ab vs [0.1] LC007	0,03	0,76	>0,05	0,85	>0,05	0,94
no Ab vs [1] LC007	>0,05	1,13	>0,05	1,06	>0,05	1,16
no Ab vs [10] LC007	>0,05	1,01	>0,05	0,87	>0,05	1,01
[0.1] LC007 vs [1] LC007	0,01	1,49	0,01	1,25	>0,05	1,24
[0.1] LC007 vs [10] LC007	0,03	1,34	>0,05	1,02	>0,05	1,08
[1] LC007 vs [10] LC007	>0,05	1,11	0,01	0,82	>0,05	0,87
no Ab vs [0.1] LC007GE	0,002	2,88	0,002	1,61	>0,05	1,03
no Ab vs [1] LC007GE	<0,001	4,53	<0,001	3,17	>0,05	1,58
no Ab vs [10] LC007GE	<0,001	5,73	<0,001	3,10	0,04	1,53
[0.1] LC007GE vs [1] LC007GE	0,02	1,57	<0,001	1,98	>0,05	1,53
[0.1] LC007GE vs [10] LC007GE	<0,001	1,99	0,002	1,93	>0,05	1,48
[1] LC007GE vs [10] LC007GE	0,05	1,27	>0,05	0,98	>0,05	0,97

	no Ab		[0.1] LC007		[1] LC007		[10] LC007		[0.1] LC007GE		[1] LC007GE		[10] LC007GE	
	p-value	fold diff.	p-value	fold diff.	p-value	fold diff.	p-value	fold diff.	p-value	fold diff.	p-value	fold diff.	p-value	fold diff.
VH vs FL	<0,001	1,95	>0,05	1,58	0,002	1,89	0,01	1,96	<0,001	5,44	<0,001	5,61	<0,001	7,34
VL vs FL	<0,001	3,69	<0,001	3,35	<0,001	3,38	<0,001	3,17	<0,001	5,74	<0,001	7,44	<0,001	7,49
VL vs VH	0,002	1,89	<0,001	2,12	<0,001	1,78	0,003	1,62	>0,05	1,05	0,02	0,76	>0,05	1,02

Figure 3C

	CD16-VH-CAR	CD16-VL-CAR	CD16-FL-CAR	CD16-del
	p-value	p-value	p-value	p-value
no Ab vs Cetuximab	<0,001	<0,001	0,003	>0,05
no Ab vs LC007	>0,05	>0,05	>0,05	>0,05
no Ab vs LC007GE	<0,001	<0,001	0,002	>0,05
LC007 vs Cetuximab	<0,001	<0,001	0,003	>0,05
LC007 vs LC007GE	<0,001	<0,001	0,002	>0,05
LC007GE vs Cetuximab	>0,05	>0,05	>0,05	>0,05

Figure 4B

	LC007						LC007 GE						no Ab					
	without vs with PBMC		without vs with IVIg 10		without vs with IVIg 1		without vs with PBMC		without vs with IVIg 10		without vs with IVIg 1		without vs with PBMC		without vs with IVIg 10		without vs with IVIg 1	
	p-value	fold diff.	p-value	fold diff.	p-value	fold diff.	p-value	fold diff.	p-value	fold diff.	p-value	fold diff.	p-value	fold diff.	p-value	fold diff.	p-value	fold diff.
CD16-VH-CAR	0,03	0,30	<0,001	1235,40	0,03	2,10	0,01	0,20	0,03	67,90	>0,05	0,90	>0,05	0,19	0,01	318,60	>0,05	0,32
CD16-VL-CAR	>0,05	0,87	0,001	203,78	0,01	2,82	0,01	0,09	0,002	16,98	>0,05	1,17	>0,05	0,65	>0,05	189,21	>0,05	2,46
CD16-FL-CAR	>0,05	0,89	0,04	2,72	>0,05	0,79	>0,05	0,69	>0,05	1,78	>0,05	0,76	>0,05	1,62	>0,05	1,33	0,04	0,44
CD16-del	>0,05	0,85	>0,05	1,53	>0,05	0,70	0,03	2,42	>0,05	0,75	>0,05	0,73	>0,05	1,03	>0,05	1,33	>0,05	1,16

Figure 4C

	LC007		LC007 GE		no Ab	
	without vs with PBMC	without vs with IVIg 10	without vs with PBMC	without vs with IVIg 10	without vs with PBMC	without vs with IVIg 10
	p-value	p-value	p-value	p-value	p-value	p-value
CD16-VH-CAR	>0,05	0,004	<0,001	>0,05	0,05	<0,001
CD16-VL-CAR	>0,05	0,002	0,003	>0,05	0,02	<0,001
CD16-FL-CAR	>0,05	>0,05	>0,05	>0,05	<0,001	<0,001
CD16-del	>0,05	>0,05	<0,001	>0,05	<0.05	0,01

Supplementary Figure 1

	CD16-VH-CAR		CD16-VL-CAR		CD16-FL-CAR	
	p-value	fold diff.	p-value	fold diff.	p-value	fold diff.
no Ab vs LC007	>0,05	1,17	>0,05	0,84	0,004	0,67
no Ab vs LC007GE	<0,001	2,72	<0,001	2,60	>0,05	0,66
